# Supplementary material for: The Impact of Heart Rate Variability Biofeedback on Anxiety Reduction and Batting Performance Enhancement in Taiwan University Baseball Players
Source: J Funct Morphol Kinesiol. 2025 Feb 13;10(1):65. doi: 10.3390/jfmk10010065 (PMC11843864; doi:10.3390/jfmk10010065)
Supplement: Supplementary file 1 [file jfmk-10-00065-s001.zip › File S1. Questionnaire.pdf]

The correlation between the physiological signal of baseball hitter and the performance of the strike

Thank you for participating in this study. Please help fill in the following basic information and two questionnaires.

Basic information

1. Gender： ☐Male ☐Female
2. Age：

Competitive State Anxiety Inventory – 2

A number of statements that athletes have used to describe their feelings before competition are given below. Read each statement and then choose the appropriate number to the right of the statement to indicate *how you feel right now*-at this moment. There are no right or wrong answers. Do *not* spend too much time on any one statement, but choose the answer which describes your feelings *right now.*

	Not at all	Somewhat	Moderately so	Very much so
	1	2	3	4
1. I am concerned about this competition.	☐	☐	☐	☐
2. I feel nervous.	☐	☐	☐	☐
3. I feel at ease.	☐	☐	☐	☐
4. I have self-doubts.	☐	☐	☐	☐
5. I feel jittery.	☐	☐	☐	☐
6. I feel comfortable.	☐	☐	☐	☐
7. I am concerned that I may not do as well in this competition as I could.	☐	☐	☐	☐
8. My body feel tense.	☐	☐	☐	☐
9. I feel self-confident.	☐	☐	☐	☐
10. I am concerned about losing.	☐	☐	☐	☐
11. I feel tense in my stomach.	☐	☐	☐	☐
12. I feel secure.	☐	☐	☐	☐

13. I am concerned about choking under pressure.	☐	☐	☐	☐
14. My body feels relaxed.	☐	☐	☐	☐
15. I'm confident I can meet the challenge.	☐	☐	☐	☐
16. I'm concerned about performing poorly.	☐	☐	☐	☐
17. My heart is racing.	☐	☐	☐	☐
18. I'm confident about performing well.	☐	☐	☐	☐
19. I'm concerned about reaching my goal.	☐	☐	☐	☐
20. I feel my stomach sinking.	☐	☐	☐	☐
21. I feel mentally relaxed.	☐	☐	☐	☐
22. I'm concerned that others will be disappointed with my performance.	☐	☐	☐	☐
23. My hands are clammy.	☐	☐	☐	☐
24. I'm confident because I mentally picture myself reaching my goal.	☐	☐	☐	☐
25. I'm concerned I won't be able to concentrate.	☐	☐	☐	☐
26. My body feels tight.	☐	☐	☐	☐
27. I'm confident of coming through under pressure.	☐	☐	☐	☐

Coping self-efficacy Scale

When things are not going well for you, or when you are having problems, how confident or certain are you that you can do the following:

	Cannot do at all 0	1	2	3	4	Moderately certain can do 5	6	7	8	9	Certain can do 10
1. Break an upsetting problem down into smaller parts.	☐	☐	☐	☐	☐	☐	☐	☐	☐	☐	☐
2. Sort out what can be changed, and what cannot be changed.	☐	☐	☐	☐	☐	☐	☐	☐	☐	☐	☐
3. Make a plan of action and follow it when confronted with a problem.	☐	☐	☐	☐	☐	☐	☐	☐	☐	☐	☐
4. Leave options open when things get stressful.	☐	☐	☐	☐	☐	☐	☐	☐	☐	☐	☐
5. Think about one part of the problem at a time.	☐	☐	☐	☐	☐	☐	☐	☐	☐	☐	☐
6. Find solutions to your most difficult problems.	☐	☐	☐	☐	☐	☐	☐	☐	☐	☐	☐
7. Resist the impulse to act hastily when under pressure.	☐	☐	☐	☐	☐	☐	☐	☐	☐	☐	☐
8. Try other solutions to your problems if your first solutions don’t work.	☐	☐	☐	☐	☐	☐	☐	☐	☐	☐	☐
9. Talk positively to yourself.	☐	☐	☐	☐	☐	☐	☐	☐	☐	☐	☐
10. Stand your ground and fight for what you want.	☐	☐	☐	☐	☐	☐	☐	☐	☐	☐	☐
11. See things from other person’s point of view during a heated argument.	☐	☐	☐	☐	☐	☐	☐	☐	☐	☐	☐
12. Develop new hobbies or recreations.	☐	☐	☐	☐	☐	☐	☐	☐	☐	☐	☐

13. Make unpleasant thoughts go away.	☐	☐	☐	☐	☐	☐	☐	☐	☐	☐	☐
14. Take your mind off unpleasant thoughts.	☐	☐	☐	☐	☐	☐	☐	☐	☐	☐	☐
15. Stop yourself from being upset by unpleasant thoughts.	☐	☐	☐	☐	☐	☐	☐	☐	☐	☐	☐
16. Keep from feeling sad.	☐	☐	☐	☐	☐	☐	☐	☐	☐	☐	☐
17. Keep from getting down in the dumps.	☐	☐	☐	☐	☐	☐	☐	☐	☐	☐	☐
18. Look for something good in a negative situation.	☐	☐	☐	☐	☐	☐	☐	☐	☐	☐	☐
19. Keep yourself from feeling lonely.	☐	☐	☐	☐	☐	☐	☐	☐	☐	☐	☐
20. Visualize a pleasant activity or place.	☐	☐	☐	☐	☐	☐	☐	☐	☐	☐	☐
21. Pray or meditate.	☐	☐	☐	☐	☐	☐	☐	☐	☐	☐	☐
22. Get friends to help you with the things you need.	☐	☐	☐	☐	☐	☐	☐	☐	☐	☐	☐
23. Get emotional support from friends and family.	☐	☐	☐	☐	☐	☐	☐	☐	☐	☐	☐
24. Make new friends.	☐	☐	☐	☐	☐	☐	☐	☐	☐	☐	☐
25. Do something positive for yourself when you are feeling discouraged.	☐	☐	☐	☐	☐	☐	☐	☐	☐	☐	☐
26. Get emotional support from community organizations or resources.	☐	☐	☐	☐	☐	☐	☐	☐	☐	☐	☐
27. Find solutions to your most difficult problems.	☐	☐	☐	☐	☐	☐	☐	☐	☐	☐	☐
