# Supplementary material for: The Impact of Heart Rate Variability Biofeedback on Anxiety Reduction and Batting Performance Enhancement in Taiwan University Baseball Players
Source: J Funct Morphol Kinesiol. 2025 Feb 13;10(1):65. doi: 10.3390/jfmk10010065 (PMC11843864; doi:10.3390/jfmk10010065)
Supplement: Supplementary file 1 [file jfmk-10-00065-s001.zip › Supplementary Materials.pdf]

Supplementary Materials

The questionnaires utilized in this study, including the Competitive State Anxiety Inventory-2 (CSAI-2) and the Coping Self-Efficacy Scale (CSES), are provided in the file titled "*Questionnaires.pdf*".

The CSAI-2, developed by Martens in 1990, assesses athletes' anxiety levels across three dimensions: cognitive anxiety, somatic anxiety, and self-confidence [1]. This inventory comprises 27 items and employs a 4-point Likert scale, where respondents rate how they feel at the moment using the options: "not at all," "somewhat," "moderately so," and "very much so".

The CSES measures an individual's confidence in performing coping behaviors when facing life challenges [2]. This scale consists of 26 items, each rated on an 11-point scale ranging from 0 ("cannot do at all") to 10 ("certainly can do"), reflecting the individual's perceived confidence under pressure. An additional 27th item, identical to the 6th item, was included in this study to ensure participants' attentiveness while completing the questionnaire.

References

1. Martens, R.; Burton, D.; Vealey, R.S.; Bump, L.A.; Smith, D.E. Development and validation of the competitive state anxiety inventory-2. In *Competitive Anxiety in Sport*; Human Kinetics: Chapaign, IL, USA, 1990; pp. 117–190.
2. Chesney, M.A.; Neilands, T.B.; Chambers, D.B.; Taylor, J.M.; Folkman, S. A validity and reliability study of the coping self-efficacy scale. *J. Health Psychol.* **2006**, *11*, 421–437, doi.org/10.1348/135910705X53155.
